# Challenges in rescuing snakes to protect human lives and promote snake conservation in Tamil Nadu, India

**DOI:** 10.1371/journal.pntd.0012516

**Published:** 2024-09-17

**Authors:** Anika Salim, Jarred Williams, Jose R. Almeida, Gnaneswar Chandrasekharuni, Harry F. Williams, Rajendran Vaiyapuri, Mohanraj Vaiyapuri, Rajan Viswanath, Thanigaivel Annamalai, Ketan Patel, Ponniah Thirumalaikolundusubramanian, Subaramanian Senthilkumaran, Romulus Whitaker, Sakthivel Vaiyapuri

**Affiliations:** 1 School of Pharmacy, University of Reading, Reading, United Kingdom; 2 Madras Crocodile Bank Trust, Mamallapuram, Tamil Nadu, India; 3 Toxiven Biotech Private Limited, Coimbatore, Tamil Nadu, India; 4 Urvanam Foundation, Tamil Nadu Snake Conservation Forum, Madurai, Tamil Nadu, India; 5 Ashoka Trust for Research in Ecology and the Environment, Bengaluru, Karnataka, India; 6 School of Biological Sciences, University of Reading, Reading, United Kingdom; 7 The Tamil Nadu Dr M.G.R Medical University, Chennai, Tamil Nadu, India; 8 Manian Medical Centre, Erode, Tamil Nadu, India; Universidade Nilton Lins, BRAZIL

## Abstract

**Background:**

Human-snake conflicts are common worldwide, often resulting in snakebites. Snakebite envenoming causes over 125,000 deaths and 400,000 permanent disabilities worldwide every year. India alone accounts for an average of ~58,000 annual snakebite-induced deaths. As human developments rapidly expand into suburban and rural areas, snakes are being displaced and incidences of residents finding snakes within their dwellings are increasing. Most people have an innate fear of snakes, compounded by centuries of negative influence from culture and mythology manifesting in people often attempting to kill snakes. Snake rescuers are volunteers who remove and relocate snakes to safe areas. This is a risky job that poses potentially fatal implications if bitten. These volunteers mostly receive no financial compensation for their time or transportation costs, but they choose to do it for their love of snakes, conservation, and for the altruistic nature of helping others. Snake rescuers often receive no formal training and are unfunded resulting in removing snakes improperly without adequate safety equipment or the required skill set to safely complete the task. Therefore, it is critical to determine their challenges and requirements to promote the safe rescue of snakes while protecting human lives.

**Methodology/Principal findings:**

In this study, we developed an online questionnaire and interviewed 152 snake rescuers in Tamil Nadu, India following written informed consent to determine their challenges and needs for rescuing snakes safely. The results demonstrate that most rescuers are males, and they conduct snake rescues for varying lengths of time. They mostly receive no formal training and are bitten by snakes. They spend their own money on the purchase of snake-handling equipment and on treatments if bitten or injured during a rescue.

**Conclusions/Significance:**

The rescuers highlighted the urgent need for formal training, safety equipment and standard protocols for rescuing snakes in Tamil Nadu. Overall, this study demonstrates that snake rescuing should be appropriately regulated by the authorities, in particular the Wildlife Division of State Forest Departments in India, and formal training along with necessary equipment, medical insurance and appropriate recognition should be provided to them to safely remove snakes from human dwellings and manage the safety of both snakes and humans. They can also act as educators to disseminate information about the preventive and first aid measures for snakebites as well as the ecological importance of snakes.

## Author summary

Snakebite envenoming is a high-priority neglected tropical disease that largely affects poor, rural communities living in developing countries. Human-snake conflicts are a key trigger for snakebites, and they occur mainly due to urbanisation and the rise in pest populations leads to the migration of snakes into human-dominated environments. The removal of snakes and release of them into safe habitats is critical in mitigating the snakebite crisis and saving snakes. Snake rescuers play important roles in saving snakes and humans by safely removing snakes and often educating communities about the dangers of snakes and appropriate dos and don’ts for snakebites. However, they face numerous challenges in rescuing snakes and most of their issues have not been brought to the attention of relevant authorities. In this study, we interviewed 152 snake rescuers in Tamil Nadu and highlighted their experiences, challenges and requirements in performing this task. The data demonstrate that most rescuers need certified training, recognition and identity cards from the government authorities, financial support for purchasing snake handling equipment and medical insurance for treatments if bitten by snakes and encouragement for them to be educators for snake conservation and snakebite mitigation. This study highlights the key challenges in rescuing snakes and appropriate recommendations to promote the safe rescue of snakes and the peaceful coexistence of snakes and humans.

## Introduction

Snakebite envenoming (SBE) is a high-priority neglected tropical disease that is estimated to cause around 125,000 deaths and 400,000 permanent disabilities worldwide every year [1–3]. India is the capital of this global issue, with an average of 58,000 annual deaths [4]. The ‘big four’ snakes including Russell’s viper (*Daboia russelii*), Indian cobra (*Naja naja*), saw-scaled viper (*Echis carinatus*) and common krait (*Bungarus caeruleus*) are regarded as being responsible for the most bites and fatalities in India [5,6]. However, there are over 350 snake species identified in India [6] of which around 60 are venomous and 15 are known to cause human fatalities. The climate in India provides perfect conditions for reptiles to thrive, but the high abundance of snakes partly due to the abundant rodent populations in the agricultural environment and the largest (around 1.45 billion [7]) human population of any country results in frequent human-snake conflicts. The rise in pests combined with the relatively rapid movement of humans into snake habitats often results in snakes being found inside people’s houses, among livestock or near dwellings [8]. In the summer, houses are often cooler than outside, providing a suitable place for snakes to stay cool. In the winter, the houses are warmer than outside, providing warmth and comfort from the cold for snakes. Food scraps, waste, open sewage, and firewood stacks amongst others attract small rodents, frogs, and toads to human dwellings, which in turn attract snakes [9,10]. Snakes are an effective tool for pest control [11], but a venomous snake near humans is a high-risk situation. Snakes often have a negative connotation among rural communities worldwide [10]. For centuries people have been taught that snakes bring death and misfortune [12–14]. Poor understanding and knowledge of venomous and non-venomous snake species and their ecological importance result in all snakes, even harmless species being regarded as dangerous and requiring rapid removal (mostly killing) [15,16]. Some snakebites occur when attempting to remove or kill the snake as the snake feels threatened and will attempt to defend themselves via biting. In recent years, through education and public awareness activities, several rural communities have been taught that there are safer, less detrimental ways to remove snakes from their homes [15–17]. Notably, most bites happen to labourers in rural communities while walking at night without a torch or protective footwear around bushes and their crops.

Snake rescuers are becoming increasingly common worldwide, and they mostly perform rescues voluntarily [18]. They face numerous challenges and lack formal training and recognition from the authorities and the public. For example, snake removals are risky as often the snakes are stressed and feel threatened resulting in a high chance of being bitten [19]. Snake rescuers require an in-depth knowledge of snake species in their area of operations, their behaviours and how to safely handle potentially deadly snakes [18]. Despite the associated risks, many snake rescuers do not charge any money for removals and often cover large distances to reach callouts without assistance for transportation costs. Currently, there are no recognised qualifications or training requirements to become a snake rescuer in most places. Therefore, many rescuers are putting themselves at risk, performing snake removals without appropriate training and safety equipment. In addition, while most snake rescuers are sensible and perform this service for selfless reasons, some are utilising the opportunity to gain recognition, celebrity status and popularity on social media [18]. Videos of people free handling and performing dangerous acts with venomous snakes are not uncommon and are a poor influence on many who see this and wish to replicate it which has resulted in several deaths. These risks can be mitigated with appropriate training, expertise and handling equipment. Therefore, it is important to gauge the views of snake rescuers and determine the key barriers to performing their work to develop evidence-based strategies for safely rescuing snakes, saving lives, keeping communities safe and conserving a natural ecological balance. In this study, we interviewed 152 snake rescuers in Tamil Nadu, India to establish their knowledge of snakes, challenges, and needs for effective and safer snake rescuing. These data demonstrate several key challenges and an urgent need to develop authorised training and standard operating procedures for snake rescuers in Tamil Nadu.

## Materials and methods

### Ethics statement

This study was approved by the institutional ethical committees at the University of Reading (reference number: 23/05) and Toxiven Biotech Private Limited (reference number: 2022/001).

### Study questionnaire

The questionnaire was developed with around 100 questions encompassing different categories such as demographics, background as snake rescuers, training they received and requirements, knowledge on snakebite prevention, frequency and equipment usage, safety measures, snakebite incidents and challenges and improvements required. The questions were developed in English, validated internally by the authors, and then added to an online survey platform (JISC). The online questionnaire was thoroughly checked for accuracy, flow and easy accessibility, and pre-tested by the authors and selective rescuers. Then the questionnaire was validated by 20 snake rescuers and 20 non-snake rescuers who were experts in snakebites in Tamil Nadu. All the feedback was addressed, and the final online questionnaire was developed.

### Public involvement statement

Due to the nature of this study specifically for snake rescuers, the members of the public were not involved in the design of this study, data collection and analysis and writing of this manuscript. All rescuers provided written consent to collect data and publish them in scientific journals. Participation in this study was voluntary and rescuers could withdraw their consent at any time during this study. The involvement in this study did not result in any implications for their routine practice. We will disseminate the results of this study to participants and wider communities through scientific publications, which might be followed by press releases in media in the local language (Tamil) and English, and through our established network.

### Study area

This study was conducted in Tamil Nadu, a large South Indian state with a high burden of snakebites. According to government statistics, Tamil Nadu has around 52% of its population living in rural areas. Moreover, the authors have performed various studies relating to snakebites in this state previously and established a network of different stakeholders including the Tamil Nadu Snake Conservation Forum for this study. Therefore, they selected this state for this study that was conducted in Tamil which the main authors can speak fluently.

### Data collection

The questionnaire was initially used to interview (by a trained study team) a network of snake rescuers in Tamil Nadu through a direct/face-to-face rescuers’ meeting at Erode. Most of these rescuers are associated with our social media group for the Tamil Nadu Snake Conservation Forum, and the remaining rescuers were identified through members of this forum. Currently, there is no formal registration process for voluntary snake rescuers with the government authorities in Tamil Nadu. Further interviews were then conducted via phone calls to the rescuers who did not attend this meeting with the study team. The rescuers were also encouraged to ask the other rescuers who were unavailable in the meeting to participate in this study by contacting the study team through phone calls. Overall, the data were collected from 152 snake rescuers across Tamil Nadu, India following informed written consent which was acquired before participation in this survey through direct and phone interviews. This survey was conducted between April—August 2022. The team ensured that there were no duplicate entries by checking the personal details of all the rescuers, and there were no follow-up calls with the rescuers. Anyone who was confirmed as a rescuer by experienced rescuers who are available in the conservation forum has been included in this study. Only snake rescuers over the age of 18 were allowed to participate in this study and consented. We excluded anyone who has not been rescuing snakes but wanted to become a rescuer.

### Statistical analysis

All the data were anonymised by removing personal details such as names and addresses of the participants before the analysis. All statistical analyses were performed using SPSS (IBM, USA) and R (Lucent Technologies, USA) to evaluate the association between the snake rescuers and snakebites. The analyses focussed on the association between the number of characteristics of the rescuers and the number of snakebites received. The data suggested that the number of snakebites had a very skewed distribution, with a large group receiving no snakebites. As a result, this outcome was categorised for analysis into three groups: 0, 1–4, and 5 or more snakebites. All characteristics of the snake rescuers were categorical. As a result, the association with the number of snakebites (on a categorical scale) was assessed using the Chi-square test. The exception was for the ability to recognise venomous snakes, where Fisher’s exact test was preferred due to the small numbers in one of the groups. A p-value of <0.05 was considered the statistically significant difference between groups.

## Results

### Demographics of snake rescuers

**Fig 1:**
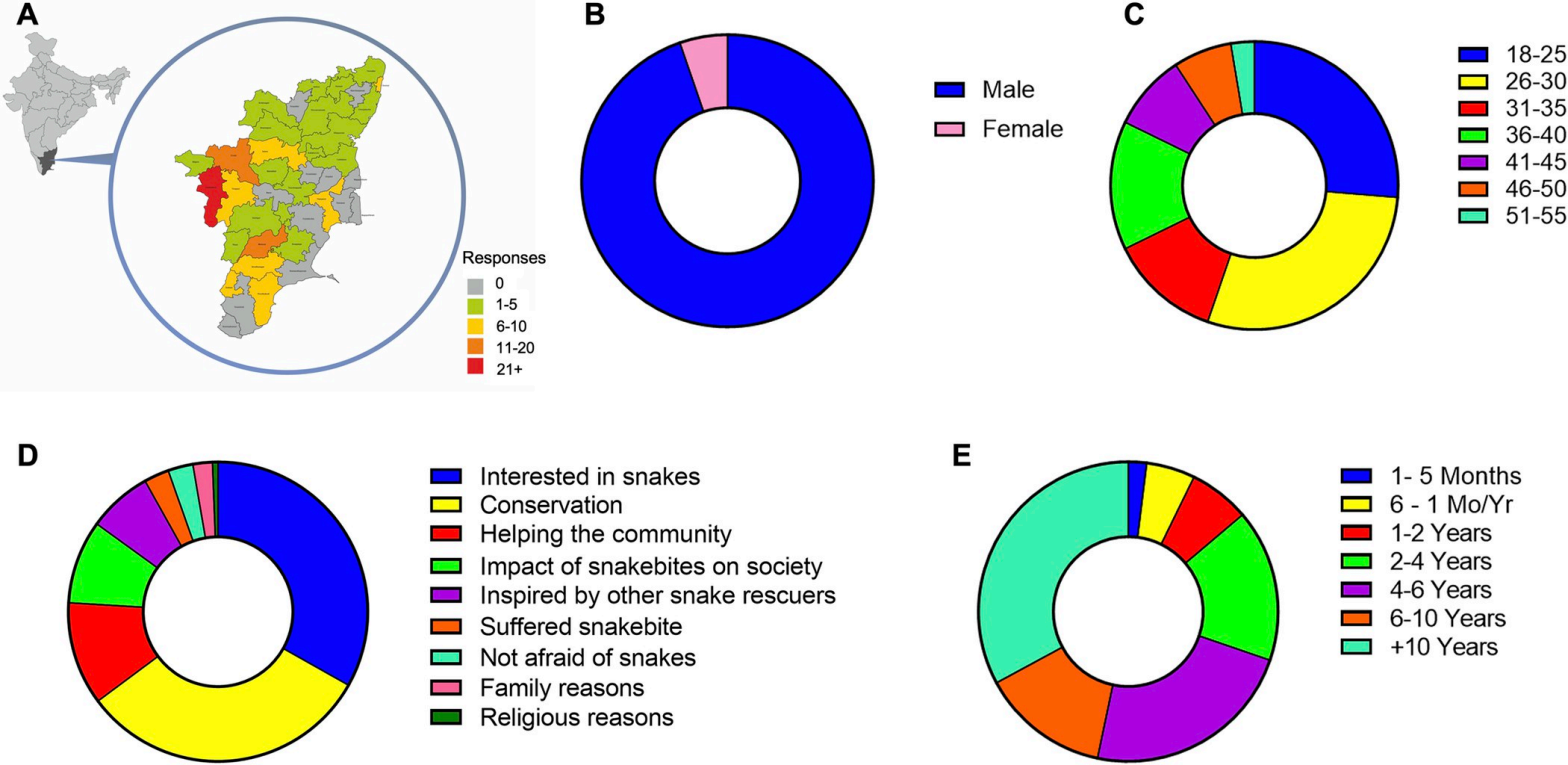
Demographic data of snake rescuers who participated in this study. **A)** A geographical heatmap displaying the responses that we received from snake rescuers in different districts of Tamil Nadu, India. This map was drawn by the authors based on the freely available political maps of India (from Survey of India - https://surveyofindia.gov.in/pages/political-map-of-india). The gender (**B**) and age groups (**C**) of snake rescuers are shown. Similarly, their reasons for rescuing snakes (**D**) and the length of the period involved in this task (**E**) are shown.

In total, 152 snake rescuers were interviewed following written informed consent using an online questionnaire. The interviewees were from a wide geographical distribution across Tamil Nadu. The responses were received from 27 out of the 38 districts in Tamil Nadu (**Fig 1A**). Notably, 24.7% of respondents were from the Coimbatore District, 11.3% from the Erode District and the same percentage were from the Madurai District. The remaining 52.7% of participants were distributed throughout the other 24 out of 38 districts within Tamil Nadu. Of the 152 snake rescuers, most of them were males [144 (94.7%)] and only a small number of them were females [8 (5.3%)] (**Fig 1B**). Most rescuers were from the younger age groups [40 (26.3%) in 18–25 and 44 (28.9%) were in 26–30], and only a small number of rescuers were in other age groups (**Fig 1C**). No responses were received from people aged over 55 years indicating the high involvement of youngsters in performing snake rescues. The majority of rescuers [110 (72.4%)] had received a tertiary (university level) education, while most others have secondary level education (6^th^ to 12^th^ standard). One participant had primary school education (1^st^ to 5^th^ standard), three had either technical/vocational training and one received no formal education. A small [14 (9.2%)] proportion of rescuers had a primary occupation in fire and rescue service, 13 (8.6%) were engaged in their own business, nine (5.9%) were working as engineers or technicians and five (3.3%) were engaged in agriculture, while others were engaged in different occupations. Most rescuers indicate that they perform snake rescues due to their interest in snakes [110 (72.4%)] and/or their conservation [105 (69.1%)] (**Fig 1D**). The other reasons for them rescuing snakes were 1) to help communities, 2) due to the impact of snakebites on society, 3) inspired by other snake rescuers, 4) suffered from snakebites themselves, 5) having no fear of snakes, and 6) due to family or religious reasons. Many of the rescuers [50 (32.9%)] had been involved in snake rescues for 10+ years, while others were involved in this task for varying periods ranging from a few months to 10 years (**Fig 1E**). Over half [82 (54%)] of the participants mentioned that they are associated with the Department of Environment, Climate Change and Forests, Government of Tamil Nadu as snake rescuers and 29 (19%) are employed by the government in an official capacity. 75 (49.3%) rescuers are associated with non-governmental organisations (NGOs) and others are working independently. Interestingly, 127 (83.6%) rescuers are also involved in conserving other animals. These data indicate that young males are mostly involved in snake rescues with a good level of education, and there are several personal reasons for them performing this task.

### Snake rescuers require better training in practical skills and theoretical knowledge

Snake rescuers should be confident in identifying the species of snake that they are rescuing and determine whether the species is venomous or not. Nearly all [145 (95.4%)] rescuers stated they were confident in differentiating venomous and non-venomous snakes in their regions. The majority [93 (61.2%)] of participants received education on how to rescue both venomous and non-venomous snakes. Four rescuers received education for only non-venomous snake rescue and three received only for venomous snake rescue. The remaining participants did not receive any education on snake rescues. 68 (44.7%) participants received education for both venomous and non-venomous snakes and obtained both practical and theoretical training for rescuing non-venomous snakes. Some [21 (13.8%)] rescuers received only practical training and three received only theoretical training for rescuing non-venomous snakes. 58 (38.1%)] of these were trained by herpetologists or snake handling specialists, 36 (23.7%) were trained by other snake rescuers, 16 (10.5%) were trained by their friends, 15 (9.9%) by ecologists and others were trained by various means including self-training (e.g. using videos disseminated through social media), by their parents, fire and rescue officials, and veterinary care providers. For handling venomous snakes, 62 (40.8%) participants received both practical and theoretical training, 20 (13.2%) received only practical training and eight (5.3%) received only theoretical training. Regarding training, 59 (38.8%) were trained by herpetologists or snake-handling specialists, 33 (21.7%) were trained by other snake rescuers, 19 (12.5%) were trained by their friends, 11 (7.2%) were trained by ecologists and others were trained by themselves, fire and rescue officials or veterinary care providers. For non-venomous snakes, 51 (33.6%) of these participants trained others. Many [45 (29.6%)] participants train others for handling venomous snakes. Several (48) of these rescuers would like both practical and theoretical training, 10 would like only practical training and others stated only theoretical training on venomous snake handling was required. Similarly, 49 rescuers also required both practical and theoretical training, 9 needed only practical training and 3 required only theoretical training for non-venomous snake rescue. Hence, most participants [85 (56%)] needed additional training for effective and safe handling of venomous and non-venomous snakes although the remaining rescuers felt they did not need any additional training.

### Working practices vary widely between rescuers

**Fig 2:**
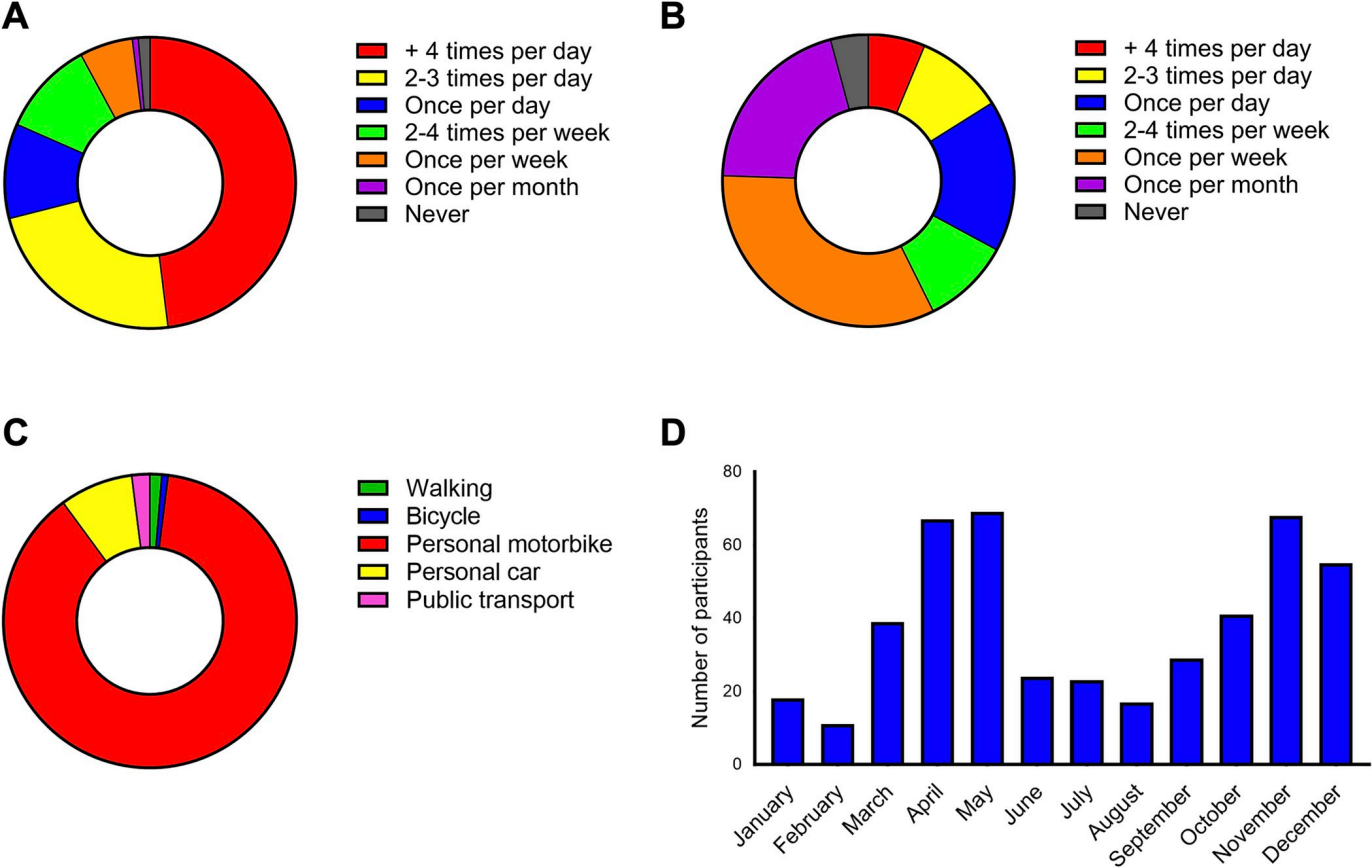
The frequency of snake rescues performed by the participants. The frequency of calls received during peak (monsoon: June/July to November) (**A**) and other seasons (**B**) are shown. (**C**) The mode of transport used to reach the location of snake rescue. (**D**) The months of the year in which most snakes were rescued by the snake rescuers.

Many snake rescuers [96 (63.2%)] were mainly contacted through the fire and rescue service. 51 (33.6%) rescuers were contacted by in-person messengers, 42 (27.4%) received phone calls from members of the public, 37 (24.3%) received phone calls from some local authorities and 11 (7.2%) were contacted through social media. Notably, rescuers were contacted by more than one route on several occasions. 73 (48%) rescuers received more than four calls per day, 35 (23%) received 2–3 calls per day, 16 (10.5%) received 2–4 times per week and others received less frequent calls during peak seasons (usually monsoon period; June/July to November) (**Fig 2A**). During non-peak seasons, 9 (6.3%) rescuers were called more than 4 times per day, 14 (9.8%) were called 2–3 times per day, 22 (15.4%) were called once per day and others received varying numbers of calls (**Fig 2B**).

Several participants [66 (43.4%)] work alone when they rescue snakes. However, 39 (25.7%) stated that they work alone sometimes but mostly they will have an assistant to aid with the removal. Notably, 46 (30.3%) rescuers never work alone as they always have an assistant with them. 107 (70.4%) rescuers stated that they are unable to rescue snakes all the time. For example, 99 (65.1%) rescuers stated that due to their work commitments, they were sometimes unable to attend the rescue calls. Some [35 (23%)] rescuers were unable to attend the rescue calls due to the distance of the location of the rescue from their homes. Some [31 (20.4%)] rescuers were unable to attend due to time constraints, 16 (10.5%) couldn’t do so due to family commitments, 15 (9.9%) due to lack of transport, and 15 (9.9%) due to lack of money. Others had various reasons including the lack of equipment, misunderstandings of the locations, and lack of assistance, due to the cancellation of services and high volume of snake rescue calls.

### Most rescuers use their own transport and complete the rescues in less than an hour

The most used mode of transport to attend snake rescue calls was personal motorbikes [131 (86.2%)] (**Fig 2C**). Others used cars, public transport, bicycles and walking to reach the location of snake rescue. The majority [92 (60.5%)] of rescuers stated that it took them on average between 16–30 minutes to travel to the location of the snake rescue, while others took longer. It is important to note that travel to a snake rescue site is completely up to the discretion of the rescuer in question and there is no obligation on their part to travel longer distances. Moreover, there are no standard guidelines to inform the maximum allowed travel distances for rescuers to rescue snakes. For non-venomous snakes, 81 (53.3%) rescuers took on average under 10 minutes, 50 (32.9%) took between 10–20 minutes, 18 (11.8%) took 21–30 minutes and others took more than 30 minutes to complete the rescues. For venomous snakes, 49 (32.2%) took under 10 minutes, 69 (45.4%) participants took 10 to 20 minutes, 23 (15.1%) took 21 to 30 minutes, and in other cases, it took longer. Following the successful removal of snakes, 128 (84.2%) rescuers stated that they did not provide the snakes (dead or alive) to the callers or witnesses present as they may kill them. However, 19 (12.5%) rescuers had to hand over the snakes to the local people due to their pressure.

Most rescuers [104 (68.4%)] had rescued over 50 snakes, 8 (5.3%) rescued 41–50 snakes, 5 (3.3%) rescued 31–40 snakes, 11 (7.2%) rescued, 21–30 snakes, 14 (9.2%) rescued between 11–20 snakes, and 10 (6.6%) rescued 1–10 snakes in the last one year. 61 (40.1%) rescuers mostly rescued venomous snakes, while 56 (36.8%) rescued mostly non-venomous snakes and 35 (23%) stated there was no difference between the number of venomous and non-venomous snake rescues that they performed. The number of snakes rescued across different months varied widely (**Fig 2D**). Most rescuers [145 (95.4%)] released the snakes into safe habitats and informed the forest department. There are no standard guidelines on where and how the rescued snakes should be released. Therefore, the rescuers will attempt to release the snakes in what they consider ‘safe’ locations that are distant from human habitats and inform the forest department. One rescuer killed the snake and seven handed over the live snakes to the forest department. Most [88 (57.9%)] rescuers reported that they maintain accurate records of snake removals and others did not maintain any records. A few rescuers passed the information to their seniors or the forest department officials.

### Snake rescuers take various measures to ensure their safety

Most [117 (77%)] rescuers stated they avoid distraction and stay focused during the rescues to alleviate any potential harm to the snakes and themselves. Moreover, 93 (61.2%) rescuers said that they were aware of spectators and kept their distance, 82 (53.9%) were able to read the snake’s body language including stress levels, 75 (49.3%) kept a snake bag/container ready, 63 (41.4%) knew where to grab the snake, 57 (37.5%) used tools such as boots and tongs and 39 (25.7%) made their presence known to the snakes. Notably, 39 (25.7%) rescuers did not display the snakes to the public once captured. If indoors, 37 (24.3%) rescuers vacated the people first, and 19 (12.5%) moved large objects or furniture out of the room. If outdoors, 33 (21.7%) cleared the stockpiles, woods and bricks that are kept near the houses. Only around half [81 (53.3%)] of the rescuers kept an emergency number to call in case of being bitten, whilst others did not have any emergency contact details. Notably, 94 (61.8%) rescuers stated they know how to provide first aid in the event of snakebites. Importantly, 133 (81.1%) rescuers educate people during the rescue on the dos and don’ts of snakebites if they are bitten.

### Snake rescuers often experience snakebites and spend their money on treatments

Half [76 (50%)] of the participants never had a snakebite incident during their rescues, however, 41 (27%) had non-venomous snakebites only, 20 (13.2%) stated they had venomous snakebites only and 13 (8.6%) had been bitten by both venomous and non-venomous snakes. For non-venomous bites, 11 (7.2%) had been bitten only once and others had more than one bite. All of them spent between INR 1000 (~US$12) and 5000 (~$60) for their hospital treatments including wound management and antibiotics following non-venomous snakebites. For venomous snakebites, 10 (6.6%) have been bitten once and others had more bites. They spent between INR 1000 (~$12) and 40,000 (~$480) for their treatments each time. Most [101 (66.4%)] rescuers incurred injuries other than snakebites, including cuts, scrapes, strains, fractures, being bitten by a cat, a road traffic accident, head injury and dehydration during snake rescues.

The number of bites was classified into zero, 1–4 and 5+ bites to analyse the correlation between rescuers and their bites and compared with various parameters. The results demonstrate a statistically significant association between the time involved in snake rescues and the number of bites indicating that the longer they are involved in rescues, the more chance of getting bitten by snakes (**Table 1**). An association with the forest department, the ability to identify between venomous and non-venomous snakes and undergoing education on snake rescues were not significantly associated with the number of bites (**Table 1**). The results also suggest that there is no strong evidence to demonstrate that the type of training (practical and/or theoretical) received was associated with the number of snakebites of any type (**Table 2**).

**Table 1:** The association between the time involved in snake rescue, the association with the forest department, the ability to identify venomous snakes, education on snake rescues and the number of snakebites.

*Group*	*Category*	*Zero bites* *n (%)*	*1–4 bites* *n (%)*	*5+ bites* *n (%)*	*P-value*
*Time involved in*	<4 years	35 (76%)	7 (15%)	4 (9%)	**0.007**
*snake rescues*	4–10 years	24 (43%)	21 (38%)	11 (20%)	
	>10 years	21 (42%)	22 (44%)	7 (14%)	
*Association with*	No	37 (56%)	21 (32%)	8 (12%)	0.93
*forest department*	Yes	43 (53%)	27 (33%)	11 (14%)	
*The ability to identify venomous snakes*	No	4 (80%)	1 (20%)	0 (0%)	0.69
	Yes	74 (52%)	49 (35%)	19 (13%)	
*Education on*	No	23 (44%)	21 (40%)	8 (15%)	0.22
*snake rescues*	Yes	58 (59%)	29 (30%)	11 (11%)	

**Table 2:** The association between the type of training received and the number of snakebites. Types of training include either practical, theoretical or both.

*Training on the type of snake*	*Outcome*	*Training*	*Number of rescuers*	*Number bites* *Mean ± SD*	*P-value*
*For non-*	Non-venomous	No training	57	1.2 ± 2.6	0.57
*venomous*	bites	One type	26	1.5 ± 3.0	
*snakes*		Both types	69	1.6 ± 3.1	
*For venomous*	Venomous bites	No training	59	0.7 ± 1.8	0.06
*snakes*		One type	29	0.1 ± 0.3	
		Both types	63	0.3 ± 1.0	

To mitigate snakebite burden, 141 (92.8%) rescuers would be interested in learning more about snakebites and the appropriate first aid. Moreover, 147 (96.7%) participants would like to have snakebite awareness programmes in their areas to reduce snakebites. 130 (85.5%) rescuers agreed that village awareness programmes would be useful, and 126 (82.9%) participants thought that teaching first aid for snakebites at schools and colleges would be useful. Several rescuers recommended the use of information leaflets, posters, TV advertisements, newspaper articles, social media and other methods such as street theatre plays and social gatherings to disseminate information about snakes and snakebites.

### Snake rescuers are mostly not using appropriate equipment to handle snakes

Most [123 (80.9%)] rescuers were using custom-made snake hooks and/or sticks, 81 (53.3%) used cotton bags, 56 (63.8%) wore normal shoes, 35 (23%) used metal tongs without soft pads and some wear gloves and shin guards. In some cases, they borrow these tools from others when going for rescues. Several rescuers stated that these tools were mainly provided by their employers or NGOs. Some rescuers received financial support from their friends to purchase them. A small number of rescuers used their money to purchase some of these tools to perform snake rescues. The results for non-venomous snakebites suggested a statistically significant difference between those who did and did not use a snake bag. The number of non-venomous snakebites was lower for those using a snake bag. This group had a mean of 1.1 non-venomous snakebites per person, compared with a mean of 1.8 bites for those not using a snake bag. None of the other types of equipment were significantly associated with the number of non-venomous snakebites in this study population (**Table 3**). These data highlight that the rescuers often prefer to use snake hooks/sticks compared to tongs used in several other parts of the world.

**Table 3:** Associations between the equipment used for snake rescues and the number of snakebites.

*Equipment used*	*Number of rescuers who did not use any equipment*		*Number of rescuers used equipment*		*P-value*
	*n*	*Mean bites ± SD*	*n*	*Mean bites ± SD*	
**Non-venomous bites**					
Shoes	96	1.6 ± 2.9	56	1.2 ± 2.9	0.07
Shin guards	143	1.4 ± 2.8	9	2.4 ± 3.6	0.15
Hooks/sticks	30	1.3 ± 3.0	122	1.5 ± 2.9	0.59
Tongs	117	1.5 ± 2.9	35	1.3 ± 2.8	0.76
Gloves	122	1.2 ± 2.6	30	2.5 ± 3.8	0.05
Cotton bags	72	1.8 ± 3.2	80	1.1 ± 2.6	**0.04**
**Venomous bites**					
Shoes	95	0.4 ± 1.1	56	0.4 ± 1.6	0.12
Shin guards	142	0.5 ± 1.3	9	0.0 ± 0.0	0.13
Hooks/sticks	30	0.2 ± 0.8	121	0.5 ± 1.4	0.16
Tongs	116	0.4 ± 1.0	35	0.7 ± 2.0	0.51
Gloves	121	0.4 ± 1.3	30	0.4 ± 1.3	0.41
Cotton bags	71	0.6 ± 1.8	80	0.2 ± 0.6	0.26

### Snake rescuers face several challenges and need significant support

Some [37 (24.3%)] rescuers spend over INR 5000 (~US$ 60), 12 (7.9%) between INR 4000–5000 (~$ 50–60), 24 (15.8%) between INR 2000–4000 (~$25–50), 21 (13.8%) between INR 1000–2000 (~$10–25), and 12 (7.9%) spent less than INR 1000 (<$10) every year from their pocket to continue to provide their services. Several rescuers stated that the lack of government support [64 (42.1%)], travelling long distances [62 (40.8%)], transport costs [54 (35.5%)], lack of family support [26 (17.1%)], lack of medical and life insurance [26 (17.1%)], lack of community support [21 (13.8%)], lack of proper equipment [18 (11.8%)], risk of snakebites [12 (7.9%)], lack of education on snakes [8 (5.2%)] and inability to afford treatment if bitten [4 (2.6%)] as the most important challenges they face to provide this service. Most [120 (78.9%)] rescuers stated they require assistance with purchasing appropriate snake-handling equipment. Of these, 97 (63.8%) stated they needed snake hooks, 75 (49.3%) required boots, 75 (49.3%) needed gloves, 72 (47.4%) needed snake tongs, 71 (46.7%) needed snake bags, and 55 (36.2%) required shin guards. Moreover, 34 (22.4%) snake rescuers requested free/reduced treatment costs in case of injury or bites, 29 (19.1%) needed liability insurance cover for themselves and dependants considering the risky nature of their role, and 24 (15.7%) required easy access to medical care. 135 (88.8%) stated they would like more snake rescuers to be available closer to their village/town/city. 137 (90.1%) stated they would like the government, local politicians, and health services to recognise snake rescuers by providing licensing and identity cards. A small number [10 (6.6%)] of rescuers would like to receive monetary benefits, reimbursement of transportation costs, simply gratitude and to be treated with respect, and be issued with appropriate safety equipment. Overall, 120 (78.9%) rescuers stated better equipment and 125 (82.2%) needed better training along with recognition and identity cards from the government authorities.

## Discussion

India’s largely tropical climate, characterised by high temperatures and humidity levels, creates an optimal environment for herpetofauna [6]. A diverse range of venomous and non-venomous snake species identified in India are distributed across various geographical regions [5,6]. Rapid urbanisation driven by population growth has led to extensive encroachment into rural landscapes, converting forests and agricultural lands into living spaces for humans. The deforestation and habitat degradation disrupt the natural habitats of snakes resulting in them being displaced and forced into closer proximity to human dwellings [20]. Importantly, the high density of rodent prey in agricultural areas (which influences rodent populations) also accounts for the abundance of snakes, indicating the need for rodent control. In addition, human habitats often attract snakes due to the abundance of suitable hiding spots and water. The prevalent fear of snakes [16,21], coupled with limited public awareness regarding venomous species and SBE [22], exacerbates human-snake conflicts. This results in an elevated SBE incidence rate and associated deaths and disabilities [22]. Despite the Indian Wildlife Protection Act of 1972 which mandates the protection of animal species, including snakes, and makes killing snakes a punishable offence, people often kill snakes including non-venomous species, when they encounter them. This practice disrupts the ecosystem and rodent control [18]. Hence, removing snakes from human habitats and leaving them in safe locations are critical to mitigate human-snake conflicts and effectively reduce the SBE burden as well as to save snakes to protect the well-balanced ecosystem [8].

Snake rescuers play a crucial role in mitigating human-snake conflicts by safely removing snakes from human dwellings and relocating them to suitable habitats [18]. They often serve as volunteers and educators, dedicating their time, expertise, and money to saving snakes and preserving biodiversity. Their efforts are expected to reduce the risk of SBE and promote coexistence between humans and snakes [18]. However, there are numerous challenges associated with snake rescues although these issues were not brought to light to address them effectively [20]. For example, some volunteers recuse snakes without appropriate knowledge and skills solely due to their intention to become famous in media and social media to impress their local community, or due to their significant enthusiasm towards wildlife without appropriate basic proficiencies. Therefore, it is critical to analyse the challenges and gaps in rescuing snakes safely to protect snakes and humans. In this study, we analysed various aspects of snake rescues in rural Tamil Nadu and reported the challenges and appropriate measures to improve snake rescues. Our results demonstrate that snake rescues are mostly performed by young males in Tamil Nadu. There are only a few females involved in this activity. This could be mainly due to the nature of this work with wildlife and the risks associated as well as the restrictions posed by their family members and local communities. As this is a high-risk and often voluntary activity, there might be significant pressure from family members for not getting involved in this activity for married males as well as females and this results in only young males who are often unmarried getting involved in snake rescues due to their enthusiasm for wildlife.

Most rescuers achieved tertiary-level education and mainly performed snake rescues due to their affection for snakes and wildlife conservation. Many of them are associated with the forest department as volunteers or NGOs, and they are also involved in saving other animals. Therefore, this voluntary work parallels other endeavours in wildlife protection, where individuals contribute their time and skills to safeguarding various species and habitats. Previous studies have reported multiple activities performed by volunteers together with the forest departments to protect wildlife [23]. For example, they may participate in tiger conservation [23], and birdwatching activities to monitor avian populations [24] or engage in various initiatives to protect marine ecosystems [25]. Similarly, some volunteers assist in reforestation projects [26], habitat restoration efforts, or wildlife rescue and rehabilitation programs. Despite the diverse nature of these voluntary activities, they all share a common goal to conserve and protect wildlife and their habitats. The dedication and passion of these volunteers play a vital role in supplementing governmental and NGO’s efforts in wildlife protection, highlighting the importance of community involvement in conservation initiatives. However, they need appropriate recognition and training from relevant authorities to appreciate their efforts, fulfil their requirements and train them appropriately. This will encourage them to continue to provide their service to the communities and motivate others to get involved in such activities.

Indeed, our data demonstrate that the appropriate certified training for snake rescuers from the government authorities is not currently available across Tamil Nadu. Therefore, they are being trained by various individuals including herpetologists in some cases and specific NGOs (e.g., the Madras Crocodile Bank Trust). They are also training each other without realising the correct procedures for snake rescues. Similarly, their working practice varies widely, and there is no coordinated approach for performing and monitoring snake rescues. Hence, the appropriate training for snake rescuers is critical, as it directly impacts the safety of both the rescuers and the snakes they handle. Training provides rescuers with the essential knowledge and skills necessary for safely identifying, capturing, and relocating snakes, particularly for venomous species. Moreover, it ensures that rescuers understand the behaviour and biology of snakes, enabling them to make informed decisions during rescue operations. Additionally, training provides instruction on the use of specialised equipment and techniques, such as snake hooks and gloves and handling protocols to minimise the risk of injury to both humans and snakes [18]. Furthermore, training helps rescuers become local champions to develop effective communication and public education strategies, fostering understanding and awareness of snakes within local communities [17,27]. Ultimately, by investing in comprehensive training programs, we can ensure the professionalism and competence of snake rescuers, enhancing the effectiveness of their efforts in mitigating human-snake conflicts and promoting coexistence between humans and wildlife. In India, snake rescuer training is typically provided by various organisations dedicated to wildlife conservation and animal welfare. Some government agencies such as state forest departments (e.g., in Kerala and Karnataka) conduct training programs for snake rescuers, often in collaboration with non-profit organisations and expert herpetologists in the states. These initiatives aim to equip individuals with the necessary skills and knowledge to safely handle and rescue snakes, thereby contributing to human safety and wildlife conservation efforts. The rescuers should also be trained on record keeping and maintenance on snake rescues for future reference.

Our results also suggest that many rescuers do not use appropriate equipment for the safe handling of snakes and to protect themselves from bites. This is mainly due to their financial constraints, and often they seek support from NGOs and individuals to obtain these materials. Safety measures and appropriate equipment are critical for snake rescuers to ensure their safety and the well-being of the snakes they handle [18]. Therefore, rescuers are recommended to wear protective gear, including sturdy gloves, long pants, and closed-toe boots, to minimise the risk of bites and other injuries. Additionally, carrying a snake hook or appropriate tong is essential for safely capturing and handling snakes from a distance. Furthermore, a first aid kit with the necessary supplies such as pressure bandages and appropriate training to use them for snakebites is essential in emergencies. Although we advise shifting the bite victims to the nearest hospitals swiftly following the bites by immobilising the bitten limbs, the other injuries during rescue operations may need prompt first aid to prevent excessive bleeding, bone fracture, wound development and other complications. Therefore, appropriate financial support or the required tools should be provided to the snake rescuers by the government authorities, NGOs, or private sponsors to promote the safe rescue of snakes. Rescuers should also be trained in safely releasing venomous snakes into suitable habitats away from human populations, as often wrong locations are likely to affect the adaptation and welfare of snakes which may result in death [28–30]. Therefore, releasing snakes in suitable habitats is as equally important as the necessity to capture them. By adhering to these safety measures and utilising the correct equipment, snake rescuers can effectively carry out their duties while minimising risks to themselves and the snakes they rescue. An effort needs to be made by rescuers to help people identify the non-venomous snakes which may be encouraged to continue living in proximity to humans, which also serves the purpose of filling the niche that could potentially be filled by venomous snakes.

Governments can play a crucial role in recognising the invaluable contributions of snake rescuers and regulating the legal process of snake rescuing and monitoring. As stated above, establishing official accreditation or certification programs for snake rescuers can ensure that individuals receive proper training and adhere to standardised protocols [27]. Recognition through certification can enhance the credibility and professionalism of snake rescuers within the community and provide them with the necessary legitimacy to carry out their duties. Hence, the Government of Tamil Nadu and other Indian states can consider initiating certified training for snake rescuers across the state, as has been performed in both Kerala and Karnataka. They should also be provided with identity cards and relevant licenses to perform their duties. Additionally, governments can create regulatory frameworks and licensing systems to govern the practice of snake rescuing, ensuring that it is conducted safely and ethically. This can include guidelines for the humane handling and relocation of snakes, as well as protocols for reporting and documenting rescue activities. This will help the authorities to identify the hot spots for specific snakes and take appropriate measures to mitigate human-snake conflicts as well as the SBE burden. By formally recognising and regulating the efforts of snake rescuers, governments can promote responsible wildlife management and conservation while safeguarding public safety. In addition, SBE treatment costs can be very expensive when the rescuers seek treatment from private healthcare [31]. Therefore, the government could consider protecting them with appropriate health insurance to cover the unexpected treatment costs as they can cause serious socioeconomic ramifications to the rescuers and their families [22]. Together with NGOs and private sponsors, the governments can procure and distribute snake rescue tools to the volunteers and monitor their correct usage. Notably, the governments can introduce the significance of snakes to the ecosystem and appropriate first aid for snakebites in school curricula across the country after analysing the feasibility of this in line with local challenges and priorities in individual states.

The role of communities is also critical in mitigating human-snake conflicts and snakebites [20,27]. They can recognise and support snake rescuers through various methods, for example, by acknowledging their contributions and calling them promptly when they see the snakes. The village leaders can honour their dedication to further encourage their service to the communities. Additionally, spreading awareness about the importance of snake rescuers and the list of locally available rescuers along with their contact details to remove snakes would mitigate the killing of snakes instantly by members of the public. They should not force the rescuers to give them the snakes for killing after rescuing. The local businesses should also consider sponsoring snake rescue tools and providing some financial assistance for their travel. Furthermore, involving snake rescuers in community outreach programs or educational initiatives can help dispel myths and fears surrounding snakes while saving snakes [27]. Showing gratitude, offering practical support and avoiding any discrimination will motivate and empower snake rescuers to continue their vital work in protecting both humans and wildlife.

### Limitations of this study

This study was conducted using an online questionnaire by interviewing snake rescuers in Tamil Nadu. The responses may be slightly different if this was conducted as in-person interviews with open-ended questions as they might have provided additional details that were not captured in the questionnaire with pre-designed questions. This study was performed only in Tamil Nadu, and therefore, the information provided in this article may differ in other states of India based on their practice and settings. For example, some states provide robust guidelines for mitigating human-snake conflicts in line with the Government of India, and some states such as Kerala and Karnataka provide training and monitoring support for snake rescues. Therefore, the data presented in this study may not be the same in other states, although they would provide an approximate understanding of this situation and measures to take if they are not already in place. The information collected in this study could not be substantiated due to the lack of supporting evidence from the rescuers. Therefore, all the data presented here solely relies on the information provided by the rescuers, and thus, there might be some unexpected inaccuracies. Although we interviewed 152 rescuers in this study, the results may have been different if we included more individuals across the state. We also could not ascertain the snakebite incidents that they reported in this study and their accurate treatment costs. We also analysed only specific parameters that we thought as important, so we might have missed some other critical aspects relating to snake rescuers.

## Conclusions

Overall, human-snake conflicts and SBE can be avoided by taking appropriate steps by all relevant stakeholders. Snake rescuers play a crucial role in mitigating human-snake conflicts and promoting coexistence between humans and wildlife. Through their dedication and expertise, they safeguard both human safety and the well-being of snakes. However, their efforts are often under-recognised and under-supported by various stakeholders. Communities, governments, and NGOs must acknowledge the invaluable contributions of snake rescuers and provide them with the recognition, appropriate training, insurance and resources they need to carry out their duties effectively. By doing so, we can enhance public safety, conserve biodiversity, and foster a deeper appreciation for the intricate balance of nature. Recognising the significance of snake rescues not only protects human lives but also ensures the survival of these misunderstood creatures, ultimately contributing to a more sustainable and harmonious relationship between humans and wildlife.
